# Proprioceptive acuity is core for back awareness in chronic low back pain: Further analysis of the content validity of the Spanish version of the Fremantle Back Awareness Questionnaire

**DOI:** 10.3389/fnhum.2022.1070402

**Published:** 2023-02-17

**Authors:** Nuria García-Dopico, Alejandro de la Torre-Luque, Carolina Sitges, Olga Velasco-Roldán

**Affiliations:** ^1^Department of Nursing and Physiotherapy, University of the Balearic Islands (UIB), Palma, Spain; ^2^Research Institute of Health Sciences (IUNICS), Health Research Institute of the Balearic Islands (IdISBa), Palma, Spain; ^3^Department of Legal Medicine, Psychiatry and Pathology, CIBERSAM ISCIII, Complutense University of Madrid (UCM), Madrid, Spain; ^4^Department of Psychology, University of the Balearic Islands (UIB), Palma, Spain

**Keywords:** low back pain, body perception, Fremantle Back Awareness Questionnaire, proprioception, validity

## Abstract

Treatments aimed at increasing self-perception may improve chronic low back pain (CLBP) symptomatology and present novel management approaches. Consequently, it is important to have valid, complete, and reliable tools for its assessment, and to understand which variables influence altered back awareness. We aimed to evaluate the face/content validity of the Spanish version of the Fremantle Back Awareness Questionnaire (FreBAQ-S) among people with and without CLBP, and to explore additional variables suggested to be involved in back awareness. A total of 264 individuals with CLBP and 128 healthy controls (HC) answered an online survey, including the FreBAQ-S, and questions regarding the completeness, comprehensibility, time-to-complete adequacy, and time spent completing it. If participants declared a lack of completeness, they had to report which aspects would be incorporated into the questionnaire to explore additional back-awareness-related variables. A statistically significant difference in completeness emerged between groups (*p* < 0.01). The questionnaire was comprehensible for more than 85% of participants, regardless of the group (*p* = 0.45). CLBP participants spent significantly more time in completing the questionnaire than controls (*p* < 0.01), but no differences were found between groups regarding the time-to-complete adequacy (*p* = 0.49). Regarding the back-awareness-related variables, 77 suggestions from CLBP group and seven from the HC were received. Most of them were related to proprioceptive acuity such as posture, weight, or movement patterns, among others. The FreBAQ-S demonstrated adequate face/content validity, completeness, comprehensibility, and adequate time of response. The feedback provided will help improve currently available assessment tools.

## 1. Introduction

Chronic low back pain (CLBP) is the leading cause of years lived with disability worldwide (Abbafati et al., 2020), with prevalence ranges between 2 and 25% (Schuttert et al., 2021), mostly affecting to women and individuals aged between 20 and 59 years (Meucci et al., 2015). Altogether, between 5 and 10% of emerging cases will become chronic (Meucci et al., 2015). In Spain, CLBP imposes a huge socioeconomic burden, impacting on absenteeism, presenteeism, and excessive health services utilization (Alonso-García and Sarría-Santamera, 2020). Additionally, current interventions fail to provide a long-term pain relief (van Tulder et al., 2006; Bredow et al., 2016), emphasizing the need of discovering the underlying causes and chronicity mechanisms of CLBP (Shaw et al., 2010; Costa et al., 2013; Aoyagi et al., 2019), and encouraging its addressing through cost-effective interventions (Alonso-García and Sarría-Santamera, 2020).

Body image, or “how the body feels to its owner” has shown to be disrupted in people with CLBP (Lotze and Moseley, 2007; Wand et al., 2014). From a sensorimotor perspective, it often refers to the implicit maps that encode the position, movement, and anthropometric characteristics of the body that are the basis for motor commands (Lotze and Moseley, 2007). Thus, it is important in sensorimotor control, and is thought to be maintained by ongoing tactile, proprioceptive, and visual input (Lotze and Moseley, 2007), which are also affected by CLBP symptomatology. In CLBP, there is substantial evidence of functional (Kong et al., 2013; Mao et al., 2014; Kregel et al., 2015; Vrana et al., 2016), morphological (Seminowicz et al., 2011; Wand et al., 2011; Baliki et al., 2012; Haggard et al., 2013; Mao et al., 2013; Kregel et al., 2015; Hotz-Boendermaker et al., 2016), and neurochemical changes (Sharma et al., 2012; Wand et al., 2014) in somatosensory and motor brain areas assumed to subserve body perception (Lotze and Moseley, 2007). Perceptual dysfunction deficits have been reported in recent literature, including poor graphesthesia (Wand et al., 2010, 2014), diminished tactile acuity (Lee et al., 2010; Catley et al., 2014; Wand et al., 2014), decreased peripheral sensory stimuli processing (Moseley, 2008; Wand et al., 2010, 2013b,^2014^; Moseley et al., 2012; Boesch et al., 2016), altered sense of ownership of the back (Boesch et al., 2016), degraded proprioception (Lee et al., 2010; Wand et al., 2014), impaired lumbopelvic motor control (Luomajoki and Moseley, 2011; Boesch et al., 2016), reduced trunk motor imagery performance (Bray and Moseley, 2011; Bowering et al., 2014; Wand et al., 2014; Suso-Martí et al., 2020), and altered perceived size/shape and awareness of the back (Moseley, 2008; Wand et al., 2014; Boesch et al., 2016). Furthermore, the back is perceived as fragile and vulnerable (Bunzli et al., 2015; Darlow et al., 2015), elicit feelings of exclusion, alienation, and rejection (Crowe et al., 2010), and it is represented different when asked to draw how it feels (Moseley, 2008; Nishigami et al., 2015).

Interventions targeting body perception distortions in CLBP may present novel management approaches to improve CLBP symptomatology (Frettlöh et al., 2006; Barker et al., 2008; Wand et al., 2011, 2012, 2013a,^2014^; Ryan et al., 2014; Daffada et al., 2015; Gutknecht et al., 2015; Wälti et al., 2015). Consequently, a growing body of evidence is focused on the role of disrupted body image as a possible contributor and target for treatment (Lotze and Moseley, 2007; Moseley, 2008; Crowe et al., 2010; Wand et al., 2014, 2016). Although it still lacks a gold standard for back awareness assessment, the Fremantle Back Awareness Questionnaire (FreBAQ-S) (Wand et al., 2014, 2016) is a recently developed, quick, simple, and low-cost tool to assess back-specific altered body perception in patients with CLBP. It comprises 9 items with a five-point Likert scale, expressed as a summatory out of a maximum of 36 points, assessing neglect-like symptoms (items 1–3), proprioceptive acuity (items 4, 5), and trunk shape and size (items 6–9) (Wand et al., 2014, 2016). The questionnaire demonstrated good psychometric properties across all the validations (Wand et al., 2014; Janssens et al., 2017; Ehrenbrusthoff et al., 2018; Nishigami et al., 2018; Erol et al., 2019; Mahmoudzadeh et al., 2020; Rao et al., 2021). FreBAQ-S scores have consistently been associated with pain intensity and duration, disability, and catastrophizing (Janssens et al., 2017; Nishigami et al., 2018; Erol et al., 2019). Correlations with anxiety, depression, fear avoidance and fear of movement were also reported, although with some inconsistences between different language-validated versions (Wand et al., 2016; Nishigami et al., 2018; Erol et al., 2019). Only in the Spanish version of the FreBAQ-S, a moderate correlation with central sensitization and a weak with pain vigilance-awareness was explored and supported, suggesting a relationship between disrupted body perception and neuroplastic changes (García-Dopico et al., in press).

Although its importance, the construct “back awareness” is relatively novel in the assessment of chronic pain conditions. Thus, it is important to explore it under the view of people suffering from CLBP, which is part of the paradigm of “patient participation” (i.e., “the involvement of patients in sharing information, feelings and signs, and accepting health team instructions”) (Vahdat et al., 2014). Planning and providing patient-oriented healthcare services, based on their needs and preferences is challenging. However, it is key to reducing anxiety and dissatisfaction and enhancing satisfaction, trust, quality of life, patients empowerment, health, planning, and decision-making improvements, among others (Vahdat et al., 2014). Therefore, patient participation is being considered among people’s civil rights and good moral values (Vahdat et al., 2014), as the adoption of policies or decisions associated with health affect their lives. Thus, surveying patients’ experiences would provide valuable information (Vahdat et al., 2014), which might be fundamental in the development and improvement of adequate and comprehensive assessment tools.

The aim of this study was to assess the face/content validity of the Spanish version of the FreBAQ-S among people with and without CLBP, and to explore their suggestions about additional variables involved in back awareness. Face validity is defined as “the extent to which a test is subjectively viewed as covering the concept it purports to measure and refers to the transparency or relevance of a test as it appears to test participants” (Holden, 2010). To the best of our knowledge, patients’ perceptions regarding back awareness have not yet been thoroughly explored. Knowing what back awareness means for people with CLBP would let clinicians and researchers to better understand this construct, to improve the available assessment tools and to reach a consensus between patients and practitioners.

## 2. Methods

### 2.1. Design

The Clinical Research Ethics Committee of the Balearic Islands approved this cross-sectional observational study (Spain, 4502/21 PI). All participants provided informed consent, and procedures conformed to the Declaration of Helsinki.

### 2.2. Participants

Volunteers were recruited through posters in clinical and non-clinical settings, social media, and institutional mailing. Participants with CLBP were included if they suffered from CLBP for more than 3 months uninterruptedly and were fluent in Spanish. HC were included if they were back pain free, having no episode of back pain within the last 2 years restricting from work or leisure activities, and fluent in Spanish. Volunteers were excluded if pregnant or early post-partum, if had specific CLBP, or if presented with severe scoliosis; psychological illness (major depressive, generalized anxiety, psychotic, or bipolar disorder); a central neurological disorder; a terminal illness; substance dependence; criminal litigation, or financial compensation related to their CLBP. Finally, 264 individuals with CLBP and 128 HC were recruited. For further information, refer to Table 1.

**TABLE 1:** Sociodemographic and clinical self-reported data.

	CLBP (*n* = 264) Mean (SD)	HC (*n* = 128) Mean (SD)	Contrast test	Effect size
Sex, male (*N*, %)	91 (34.46)	34 (26.56)	4.4	0.07
Age, years	45.67 (11.59)	39.52 (12.66)	3.3**	0.5^††^
Height, meters	1.68 (0.1)	1.67 (0.08)	0.95	0.11
Weight, kg	74.5 (19.11)	65.57 (12.53)	2.58**	0.55^††^
BMI	25.23 (5.31)	23.42 (3.82)	2.37**	0.39^†^
**Working status**				
Active	213 (80.68)	114 (89.06)	3.79	0.07
On leave	16 (6.06)	0	6.61*	0.09
Retiree or pensioner	19 (7.19)	4 (3.12)	1.9	0.16
Unemployed	16 (6.06)	10 (7.81)	0.19	0.02
Months suffering pain	97.6 (103.29)	–	–	–
**Pain intensity (Numerical Pain Rating Scale, 0–10), mean (SD)**				
At the moment	5.28 (2.34)	0.57 (1.06)	17.92**	2.59^†††^
Usually	5.62 (2.01)	1 (1.36)	18.64**	2.69^†††^
At the worst moments	8.6 (1.29)	3.7 (2.77)	12.84**	2.26^†††^
**Back perception**				
FreBAQ-S	11.38 (7.27)	4.98 (4.99)	6.26**	1.03^†^
Completeness (*N*, %)	–	–	6.16**	0.91
Yes	173 (65.53)	116 (90.62)		–
No	91 (34.47)	12 (9.38)	–	–
Comprehensibility (*N*, %)	–	–	0.75	0.131
Yes	227 (85.98)	111 (86.71)	–	–
No	37 (14.02)	17 (13.29)	–	–
Time to complete (*N*, %)	2.55	1.49	4.834**	0.79

CLBP, chronic low back pain; HC, healthy controls; SD, standard deviation; BMI, body mass index; Contrast test for continuous variables is *t*-student; for binary/categorical variables is χ^2^; Effect size for continuous variables is Cohen’s d; for binary/categorical variables is Crammer’s V. FreBAQ-S, Fremantle Back Awareness Questionnaire, Spanish version. Each of the 9 items of the FreBAQ-S accounts between 0 and 4 points. Mean sum scores are reported (questionnaire range 0–36). Significance level: *p < 0.05, **p < 0.01. Effect sizes: small (≥0.2)^†^, medium (≥0.5)^††^, large (≥0.8)^†††^.

### 2.3. Data acquisition

The data were collected on a larger study undertaking the cross-cultural adaptation of the FreBAQ-S between April and October 2021 (García-Dopico et al., in press), including an extensive assessment of cognitive-affective and behavioral variables related to pain experience (pain, kinesiophobia, pain catastrophizing, depression, anxiety, stress, fear-avoidance beliefs, pain vigilance, physical activity, prognosis for CLBP, disability, and central sensitization). To consult the FreBAQ-S, see Supplementary Appendix A. It was conducted on-line to protect participants against SARS-CoV-2. Willing adult volunteers had to access to the on-line form, where were screened for eligibility and declared informed consent.

This report describes further analysis derived from the face/content validity assessment of the FreBAQ-S (Beaton et al., 2000), based on four additional questions of the FreBAQ-S (Janssens et al., 2017; Ehrenbrusthoff et al., 2018). Questions regarding completeness (“Do you think that this questionnaire covers the most important aspects of altered back related perception?”) and comprehensibility (“Are the questions sufficiently comprehensibly worded?”) should be answered “yes” or “no.” If answered “no” for completeness, participants were requested to provide qualitative feedback, suggesting variables that should be considered when assessing back awareness. This aimed to explore variables that people with CLBP feel to be related to back awareness. If participants answered “no” for comprehensibility, were asked to describe the problematic item. Furthermore, they had to rate the time to complete the questionnaire adequacy (from 0 to 10, with 0 representing “unacceptably long” and 10 “completely ok”) and the approximate time spent to complete, to add evidence on the usability of the FreBAQ-S. For further information, see Supplementary Appendix B.

### 2.4. Data analysis

Data were analyzed using Rstudio (version 4.1.1) (RStudio, 2021), with alpha level set at 0.05. All the variables were assessed for normality. Parametric and non-parametric statistics were tested on *a priori* non-normal variables. After inspection, parametric statistics are provided. Descriptive statistics by study group were performed for binary (relative frequencies) and continuous variables (mean, SD). Between-group differences were assessed for quantitative (Student’s *t*-test) and categorical variables (χ^2^).

#### 2.4.1. Face/content validity

The results about the face/content validity of the FreBAQ-S are expressed by percentages regarding completeness, comprehensibility, time to complete adequacy and approximate time to complete. Differences between groups were assessed with Student’s *t*-test and χ^2^. Qualitative feedback was inspected to identify the least understandable items. Following previous research (Janssens et al., 2017; Ehrenbrusthoff et al., 2018), a preset threshold of 50% negative responses was established to judge the need for cultural adaptations.

#### 2.4.2. Back-awareness-related variables

The qualitative feedback was accounted and summarized under the three topics of the original FreBAQ-S: neglect-like symptoms, proprioceptive acuity, and trunk shape and size. An additional topic, entitled “psychological back-awareness-related variables” was purposed for items that did not fit in the original structure. The feedback was accounted as “suggestions” for the analysis, as each participant could provide more than one. Complaints regarding rehabilitation outcomes and drug intake were excluded for not being related to the FreBAQ-S.

## 3. Results

### 3.1. Sample description

Initially, 463 volunteers showed interest. *N* = 2 participants declined consent, *N* = 39 did not meet inclusion criteria, and *N* = 30 meets any exclusion criterion and were, therefore, excluded (*N* = 71). Table 1 summarizes participants’ sociodemographic and clinical data. The CLBP group was 6.15 years older, on average (*t* = 3.3; *p* < 0.01), had a higher weight (*t* = 2.58; *p* < 0.01) and a higher body mass index (*t* = 2.37; *p* = 0.02). Statistically significant differences were found between groups regarding pain intensity in current (*t* = 17.92; *p* < 0.01), usual (*t* = 18.64; *p* < 0.01), and worst pain (*t* = 12.84; *p* < 0.01). The CLBP group also showed a significantly higher proportion of participants on sick leave of work due to pain (*t* = 6.61; *p* < 0.05). No significant differences were found among the remaining variables.

### 3.2. Face/content validity

Although both groups declared that the FreBAQ-S covers the most important aspects of altered back perception (65.53% for CLBP, 90.62% for HC), a statistically significant difference between groups emerged regarding completeness (*p* < 0.01). The questionnaire was comprehensible for the 86.22% of participants, regardless the group (*p* = 0.45; 85.98% for CLBP, 86.71% for HC). However, 10 participants with CLBP and three HC had subjective difficulties, as found the items “too subjective” and “difficult to imagine.” Item 4 was difficult to understand for 15 individuals with CLBP (5.68%) and five HC (3.9%). Double denials of items 4–6 were confusing for one CLBP participant and two HC. One participant with CLBP reported an inconsistency between items 4 and 5 and the available answers, explaining that the question refers to “the how” and the answer to “the frequency” (e.g., Item 4, “When performing everyday tasks, I don’t know how much my back is moving” – “Rarely feels like that”). Table 2 summarizes all declared issues regarding comprehensibility for both groups. No between-groups differences were found in time-to-complete adequacy (*p* = 0.49), with 52.29% of the overall participants rating it as 10/10 and 86.22% rating it ≥8/10. Participants mostly spent between 1 and 2 min (25.76%) answering the FreBAQ-S. On average, participants with CLBP spent more time than HC (*p* < 0.01). Specifically, 11.22% of participants spent more than 5 min, 93.18% of whom had CLBP.

**TABLE 2:** Comprehensibility of the FreBAQ-S between groups.

		Item (*N*, %)									
		**1**	**2**	**3**	**4**	**5**	**6**	**7**	**8**	**9**	**1–9**
**CLBP (*N* = 264)**	Item comprh.	4 (1.51)	0	4 (1.51)	15 (5.68)	4 (1.51)	3 (1.14)	2 (0.75)	2 (0.75)	0	8 (3.03)
	Item wording	1 (0.38)	0	0	0	0	0	1 (0.38)	1 (0.38)	1 (0.38)	2 (0.75)
	Subjective issues	1 (0.38)	1 (0.38)	1 (0.38)	1 (0.38)	2 (0.75)	0	2 (0.75)	0	0	2 (0.75)
	Double denial	1 (0.38)	0	0	1 (0.38)	1 (0.38)	1 (0.38)	0	0	0	0
**HC (*N* = 128)**	Item comprh.	0	0	0	5 (3.9)	2 (1.56)	1 (0.78)	0	0	0	1 (0.78)
	Item wording	0	1 (0.78)	0	1 (0.78)	0	0	0	0	0	1 (0.78)
	Subjective issues	0	0	0	0	0	0	0	0	0	3 (2.34)
	Double denial	0	0	0	1 (0.78)	1 (0.78)	0	0	0	0	2 (1.56)

CLBP, chronic low back pain; Comprh., comprehensibility; Subjective issues refers to difficulty identifying the symptoms or sensations described at the item.

### 3.3. Back-awareness-related variables

In total, 37.88% of the participants with CLBP suggested additional back-awareness-related variables, whereas only 4.69% of the HC did. The CLBP group provided 112 improvement suggestions. Of those, 26 suggestions were related to pain, specifically, the pain experience (13), pain intensity (3), kind of pain (8), and pain timing (2), and 9 were not related to the FreBAQ-S. Thus, 35 suggestions were out of the scope of the questionnaire, and were, therefore, excluded. A total of 77 valid suggestions were received from the CLBP group and are summarized, with representative verbatims, in Table 3. Mostly, the suggestions related to proprioceptive acuity (63/77), with lesser regarding neglect-like symptoms (1/77), and trunk shape and size (2/77) topics. Only seven suggestions addressed psychological back-awareness-related variables. Contrary, the HC group just provided 10 improvement suggestions. From those, one was related to pain experience and two were not related to the FreBAQ-S and were, therefore, excluded. Thus, seven valid suggestions were received from the HC group and are summarized, with representative verbatims, in Table 4. The suggestions mostly related to proprioceptive acuity, with only one focused on psychological back-awareness-related variables and neither one on neglect-like symptoms or trunk shape and size.

**TABLE 3:** Variables suggested to be related to back awareness by the chronic low back pain group.

	Number of suggestions	Representative verbatim
**Neglect-like symptoms**	1	
Alienation	1	“I feel my back is a separate organ that harms the rest of my body”
**Proprioceptive acuity**	63	
Pain-related proprioception	7	“Pain makes me know where my back is” “Pain starts at a region of the back and extends to others”
Posture	10	“I am aware of the postures I adopt” “I adopt certain postures at certain moments” “When I move I have to think about my back’s position”
Weight	5	“I feel my back as an extra weight” “I feel my back cannot handle my own weight” “When I walk my back weights more” “I feel a weight on my back”
Tingle	1	∼
Heat	1	∼
Pinching, puncture	3	“When I sit, I feel punctures” “I feel as if my back muscles are hooked with the bones and pinched”
Pressure	2	“I feel my back is under pressure”
Blockage, stiffness	15	“I feel a blockage on my lower back” “I feel my extremities stiff” “I feel like my back is going to split” “I feel like something prevents me from moving my back” “My back feels like a block, not chain of vertebra” “I feel my back is oxidized and needs to be moved” “I feel changes on the stiffness of my back” “I feel like my back muscles contract”
Movement	10	“How I feel my back affects my daily movements” “I feel my back has not enough strength to perform certain movements”
Failure/inability	8	“I feel my back can stop functioning” “I feel my lower back is weak”
Muscle fatigue	1	∼
**Trunk shape and size**	2	
Spine physiological curves	2	“I feel different my lordosis”
**Psychological back-awareness-related variables**	11	
Cognitive effort	1	“I need to focus when performing certain movements”
Attention to pain	2	“Where do you focus your thoughts when feeling pain?”
Fear	1	“Sometimes I lose the control of my back, and this scares me”
Resilience/normalization	2	“My back is the center of my daily living” “I feel living with back pain is normal, when normal should be living without pain”
Confusion	1	“My back pain makes me feel confused and with headache”
Perceived resting	4	“My back pain makes me feel confused and with headache”

∼, participant did not provide a representative verbatim.

**TABLE 4:** Variables suggested to be related to back awareness by the control group.

	Number of suggestions	Representative verbatim
Neglect-like symptoms	0	
Proprioceptive acuity	6	
Posture	4	“When I relax I adopt postures that may damage my back” “I am aware of my posture during the day” “Do you feel you lose control of your extremities while moving your back?” “I am able/I need to correct my posture”
Movement	2	“I need to crackle my back” “Do you feel that it is difficult for you to move one part of your back in relation to another (dissociation)?”
Trunk shape and size	0	
Psychological back-awareness-related variables	1	
Perceived resting	1	∼

∼, participant did not provide a representative verbatim.

## 4. Discussion

The aim of this study was to assess the face/content validity of the FreBAQ-S among people with and without CLBP and to explore additional back-awareness-related variables. The FreBAQ-S demonstrated adequate face/content validity to assess disrupted self-perception of the back in people with CLBP, based on its adequate completeness, understandability, and time to complete. This, added to its good psychometric properties (Wand et al., 2014; Janssens et al., 2017; Ehrenbrusthoff et al., 2018; Nishigami et al., 2018; Erol et al., 2019; Mahmoudzadeh et al., 2020; Rao et al., 2021), make the FreBAQ-S suitable to use in clinical settings. Additionally, Ehrenbrusthoff et al. (2018) demonstrated, based on the minimal clinically important difference, its usability in research contexts.

The face/content validity of the FreBAQ-S was only assessed in the German and Dutch versions, achieving similar results in understandability and time to complete, although with higher completeness percentages (Janssens et al., 2017; Ehrenbrusthoff et al., 2018). However, different sample sizes and baseline pain scores between the three studies could explain differences in completeness. Mostly, participants with CLBP and HC found the German and Dutch versions of the FreBAQ-S a complete and comprehensible measure, that could be completed within an appropriate time (Janssens et al., 2017; Ehrenbrusthoff et al., 2018).

Notwithstanding, we detected a significant difference in the completeness of the FreBAQ-S between healthy and CLBP individuals, supporting a lesser sense of completeness in the CLBP group (*p* < 0.01). Although the multigroup analysis previously performed on the FreBAQ-S regarding clinical condition and sex revealed a lack of measurement invariance between CLBP and HC, it is plausible that people with CLBP have a further idea of how they feel their back when dealing with pain (García-Dopico et al., in press). Thus, although “back awareness” might mean the same for CLBP and HC, the construct could have a wider interpretation for people with CLBP, due to their pain experience. Contrary, HC may base their perception of completeness on inferences about how people with CLBP would feel about their back. However, this hypothesis should be addressed in further research.

In absence of gold standard measures of body perception, it is important to consider patient perspectives. The added value of patients’ participation in healthcare includes receiving different points of view in relation to the same subject and should result in better rehabilitation (Vahdat et al., 2014). Patient participation has been associated with improved treatment outcomes in several conditions such as diabetes, rheumatic diseases, or myocardial infarction, and with enhanced compliance with secondary preventive actions (Vahdat et al., 2014). The assessment performed aimed to provide further information regarding back awareness, given its importance as a potential contributor and target for treatment in CLBP (Lotze and Moseley, 2007; Moseley, 2008; Crowe et al., 2010; Wand et al., 2014, 2016), to improve currently available assessment methods and to design better treatment strategies, which aims, in the end, to improve the management of CLBP. Previously, only the German version of the FreBAQ-S explored additional variables involved in back awareness. Unfortunately, the number of suggestions was not reported. Participants suggested covering aspects of night sleep, stair climbing, current awareness of posture, morning stiffness, and current sensory abnormalities hampering body awareness (Ehrenbrusthoff et al., 2018), that mostly emerged also in our research. Most of the suggestions reported in our study related to proprioceptive acuity (81.81% of CLBP, 85.71% of HC). The most common suggestions reported by the CLBP pain referred to feelings of blockage and stiffness of the back, followed by posture, motor control, and sensations of failure or inability, and different sensory abnormalities, while the HC focused only on posture and motor control. Those complaints are consistent with current evidence supporting a wide range of proprioceptive deficits in individuals with CLBP (Tong et al., 2017), some of which have already been reported to be related to back awareness (Moseley, 2008; Wand et al., 2011, 2013b,^2014^; Boesch et al., 2016). Neglect-like symptoms and trunk shape and size received much less attention by the CLBP group, although current evidence supports both alterations in CLBP (Moseley, 2008; Crowe et al., 2010; Boesch et al., 2016). Although it is only a hypothesis, it is plausible that those symptoms are less evident for people who has never suffered from CLBP, which might ignore its influence in the pain experience. Although scarce, these suggestions were illustrative of current evidence supporting feelings of exclusion, alienation, and rejection of the painful area in CLBP (Crowe et al., 2010; Boesch et al., 2016), and distorted representation of the back (Moseley, 2008).

A subgroup of psychological back-awareness-related variables were reported by our participants, although the original FreBAQ-S did not assess them. While “fear” and “attention to pain” emerged as relevant issues in our study, a recent systematic review identified psychological and psychosocial variables, as pain catastrophizing or kinesiophobia, as predictors for altered central pain modulation in individuals with non-specific CLBP (Subramanian and Venkatesan, 2022). Current evidence supports that maladaptive beliefs about the nature of back problems and future consequences drive behaviors that might bring about maladaptive changes in neurobiological systems that contribute to self-perception of the back (Wand et al., 2016). In fact, back awareness has correlated with a range of psychological variables. The positive correlation between back awareness and pain catastrophizing is consistent across all the validations (Wand et al., 2014; Janssens et al., 2017; Ehrenbrusthoff et al., 2018; Nishigami et al., 2018; Erol et al., 2019; Mahmoudzadeh et al., 2020; Rao et al., 2021), whereas the correlation with kinesiophobia is supported by all, except the Persian and English versions (Wand et al., 2014; Mahmoudzadeh et al., 2020). The information provided by our sample reinforce the relevance of cognitive-affective wellbeing in body perception, which has been reported in other clinical conditions (Zhao et al., 2018; Bravo et al., 2019; Rodrigues et al., 2021) and needs to be explored in further research.

The FreBAQ-S demonstrated good comprehensibility regardless the group (>85%), with higher comprehensibility percentages when compared to previous reports (77% for CLBP in Dutch version; 82.9% for CLBP and 77.1% for controls in German version). Item 4 was reported to be the least comprehensible for both CLBP (5.68%) and HC (3.9%), in line with previous literature. In the German validation, one patient reported that the double negative expressions in questions 4, 5, and 6 could be misleading, which was supported by one of our participants with CLBP and two HC (Ehrenbrusthoff et al., 2018). Any participant of previous studies highlighted item-response inconsistences, as we found in the current study. The 9 items of the questionnaire were rated as lacking comprehensibility by eight participants with CLBP (3.03%) and one in the HC group (0.78%). However, all the scores were well below the preset threshold of 50% of negative responses. Thus, the translation process revealed no obvious cultural adaptations, and the FreBAQ-S has demonstrated to be comprehensible.

Both CLBP and HC groups declared that the time needed to compete the questionnaire was adequate (*p* = 0.49), supporting the results of the German and Dutch versions. Most of our participants spent between 1 and 2 min to answer the questionnaire, although with slightly higher times for the CLBP group. A total of 44 participants spent more than 5 min to answer the FreBAQ-S, of which 41 had CLBP. The appropriateness of time to complete was nearby the 100% for the German version. Although the appropriateness of time to complete was lower in our study, the 86.22% of our overall sample rated the time-to-complete adequacy as ≥8/10, supporting the appropriateness of the questionnaire and adding evidence to the translation process and cross-cultural validity of the FreBAQ-S.

The differences evidenced in completeness between individuals with and without CLBP allowed the exploration of emerging back-awareness-related variables that will allow the enhancement of the questionnaire. However, some important aspects must be highlighted. The influence of altered self-perception in the development and persistence of CLBP remains uncertain and the nature of the studies performed until now prevents from drawing any inferences of cause and effect. Additionally, in absence of gold standard measures of body-perception, the criterion related validity of the scale is currently unknown (Wand et al., 2014). The study was performed on a convenience sample drawn from clinical and non-clinical settings. Although this could have added heterogeneity, this is, to the best of our knowledge, the study with a larger sample size exploring the face/content validity of the questionnaire. The exclusion criteria were strict to guarantee the homogeneity of the sample, but contrary our sample may not cover the wider CLBP population. Both groups were comparable for all the assessed variables, excepting age and body mass index. However, the multigroup analysis performed in our previous study revealed a lack of measurement invariance on the FreBAQ-S regarding sex (currently under review).

Overall, our results support the adequate face/content validity of the FreBAQ-S, according to previous evidence. Even considering a significant difference in completeness between CLBP and HC, the FreBAQ-S was found to be a complete and comprehensible questionnaire, with adequate time of response. Given its importance, further quantitative and qualitative-based research should assess the influence of back awareness in the development and persistence of pain and deeply explore the construct back self-perception in individuals with CLBP to improve currently available assessment tools.

## Data availability statement

Research data are not shared as are part of an ongoing study. Codes and extended results can be accessed on reasonable request to the corresponding author.

## Ethics statement

This study, that involves human participants was reviewed and approved by the Clinical Research Ethics Committee of the Balearic Islands (Spain, 4502/21 PI). The patients/participants provided their written informed consent to participate in this study.

## Author contributions

NG-D, OV-R, and CS discussed the design of the study. NG-D, CS, OV-R, ATL, NG-D, and CS guided the statistical analysis. NG-D redacted the first draft of the manuscript. All authors reviewed and improved the manuscript.
