# Supplementary material for: Proprioceptive acuity is core for back awareness in chronic low back pain: Further analysis of the content validity of the Spanish version of the Fremantle Back Awareness Questionnaire
Source: Front Hum Neurosci. 2023 Feb 17;16:1070402. doi: 10.3389/fnhum.2022.1070402 (PMC9983349; doi:10.3389/fnhum.2022.1070402)
Supplement: Supplementary file 1 [file Data_Sheet_1.docx]

Supplementary Material:
Proprioceptive deficits are core for back awareness in chronic low back pain. Further analysis of the content validity of the Spanish version of the Fremantle Back Awareness Questionnaire

# Appendix A. Fremantle Back Awareness Questionnaire, Spanish Version.

A continuación, se muestran algunas expresiones que otras personas con dolor

lumbar utilizan para describir cómo sienten su espalda. Indique si estas frases son

aplicables a cómo percibe su espalda cuando experimenta dolor, usando la siguiente

escala:

0 = Nunca la siento así.

1 = Raramente la siento así.

2 = Ocasionalmente, o algunas veces la siento así.

3 = Frecuentemente, o una cantidad moderada de tiempo la siento así.

4 = Siempre, o la mayor parte del tiempo la siento así.

**Ítems**

1. Mi espalda se siente como si no formara parte del resto de mi cuerpo.
2. Necesito enfocar toda mi atención en mi espalda para lograr que se mueva como yo quiero.
3. Siento como si mi espalda se moviera involuntariamente, sin que lo pueda controlar.
4. Cuando realizo mis tareas diarias, no sé cuánto se mueve mi espalda.
5. Cuando realizo mis tareas diarias, no sé exactamente en qué posición se encuentra mi espalda.
6. No soy capaz de percibir el contorno de mi espalda de forma exacta.
7. Mi espalda se siente más grande (hinchada).
8. Mi espalda se siente como si hubiera encogido.
9. Mi espalda se siente como si estuviera torcida (asimétrica).

# Appendix B. Face validity assessment.

After answering the Fremantle Back Awareness Questionnaire, the on-line survey included the following questions to assess face validity of the questionnaire, as previously reported in the German validation process of the FreBAQ (Ehrenbrusthoff et al., 2018):

- ***Completeness of content***: “Do you think that this questionnaire covers the most important aspects of altered back related perception? [Yes/No]”; “If “No”, which aspects would you incorporate?”
- ***Comprehensibility***: “Are the questions sufficiently comprehensibly worded? [Yes/No]”; “If “No” which items are not sufficiently comprehensible?”
- ***Time to complete adequacy***: “Is the time needed for filling in the questionnaire appropriate?” scored on a 0–10 scale with 0 representing “unacceptably long” and 10 “completely ok”.
- ***Time spent to complete***: Participants had to report the approximate time spent to complete the questionnaire by selecting one of the following alternatives:
  - Less than one minute
  - Between 1-2 minutes
  - Between 2-3 minutes
  - Between 3-4 minutes
  - Between 4-5 minutes
  - More than 5 minutes
